# *Campylobacter jejuni dsb *gene expression is regulated by iron in a Fur-dependent manner and by a translational coupling mechanism

**DOI:** 10.1186/1471-2180-11-166

**Published:** 2011-07-25

**Authors:** Anna D Grabowska, Michał P Wandel, Anna M Łasica, Monika Nesteruk, Paula Roszczenko, Agnieszka Wyszyńska, Renata Godlewska, Elzbieta K Jagusztyn-Krynicka

**Affiliations:** 1 Department of Bacterial Genetics, Institute of Microbiology, University of Warsaw, Miecznikowa 1, 02-096, Warsaw, Poland; 2 Department of Molecular Mechanisms of Mycobacterial Infections, Institute of Pharmacology and Structural Biology, 205, route de Narbonne, 31077 Toulouse cedex, France; 3 Division of Protein and Nucleic Acid Chemistry MRC Laboratory of Molecular Biology Hills Road, Cambridge, CB2 0QH, UK; 4 Department of Gastroenterology, The Medical Centre of Postgraduate Education, Marymoncka 99/103, 01-813 Warsaw, Poland

## Abstract

**Background:**

Many bacterial extracytoplasmic proteins are stabilized by intramolecular disulfide bridges that are formed post-translationally between their cysteine residues. This protein modification plays an important role in bacterial pathogenesis, and is facilitated by the Dsb (disulfide bond) family of the redox proteins. These proteins function in two parallel pathways in the periplasmic space: an oxidation pathway and an isomerization pathway. The Dsb oxidative pathway in *Campylobacter jejuni *is more complex than the one in the laboratory *E. coli *K-12 strain.

**Results:**

In the *C. jejuni *81-176 genome, the *dsb *genes of the oxidative pathway are arranged in three transcriptional units: *dsbA2*-*dsbB*-*astA, dsbA1 *and *dba*-*dsbI*. Their transcription responds to an environmental stimulus - iron availability - and is regulated in a Fur-dependent manner. Fur involvement in *dsb *gene regulation was proven by a reporter gene study in a *C. jejuni *wild type strain and its isogenic *fur *mutant. An electrophoretic mobility shift assay (EMSA) confirmed that analyzed genes are members of the Fur regulon but each of them is regulated by a disparate mechanism, and both the iron-free and the iron-complexed Fur are able to bind *in vitro *to the *C. jejuni *promoter regions. This study led to identification of a new iron- and Fur-regulated promoter that drives *dsbA1 *gene expression in an indirect way. Moreover, the present work documents that synthesis of DsbI oxidoreductase is controlled by the mechanism of translational coupling. The importance of a secondary *dba-dsbI *mRNA structure for *dsbI *mRNA translation was verified by estimating individual *dsbI *gene expression from its own promoter.

**Conclusions:**

The present work shows that iron concentration is a significant factor in *dsb *gene transcription. These results support the concept that iron concentration - also through its influence on *dsb *gene expression - might control the abundance of extracytoplasmic proteins during different stages of infection. Our work further shows that synthesis of the DsbI membrane oxidoreductase is controlled by a translational coupling mechanism. The *dba *expression is not only essential for the translation of the downstream *dsbI *gene, but also Dba protein that is produced might regulate the activity and/or stability of DsbI.

## Background

*Campylobacter jejuni *is a human pathogen and the leading cause of acute bacterial gastroenteritis. As a commensal organism for many warm-blooded animals, especially in the gastrointestinal tract of poultry, *C jejuni *is also isolated from a wide variety of watery environmental sources [1,2]. Thus, the ability of *C. jejuni *to sense and respond to diverse environmental stimuli and to adapt gene expression to changes in external conditions is crucial for its pathogenesis, commensalism and survival outside the host organism.

Recent experiments have revealed many changes in the *C. jejuni *transcriptome and proteome that are driven by environmental stimuli. These include temperature, oxygen tension, iron concentration, sodium deoxycholate concentration and pH of the culture medium [3-7]. *C. jejuni's *phase of life - planktonic vs biofilm - also shows a great difference in the microorganism's protein profile [8,9]. *Campylobacter *gene expression is coupled to environmental cues mostly by two-component signal transduction systems (TCSTS) [10-14]. The activity and the amount of a specific protein can also be affected by posttranslational modifications such as glycosylation, proteolysis and disulfide bond formation. That latter protein modification, which very often influences the tertiary and quaternary structure of virulence determinants, plays an important role in bacterial pathogenesis [15,16]. In Gram-negative bacteria disulfide bond formation is facilitated by the Dsb (disulfide bond) family of redox proteins, which function in the periplasmic space under oxidizing conditions. In *E. coli *the disulfide bridge formation system operates in two partially coinciding metabolic pathways: the oxidation (DsbA and DsbB) pathway and the isomerization/reduction (DsbC and DsbD) pathway. The oxidation pathway is responsible for the formation of disulfide bonds in newly synthesized proteins, just after they cross the cytoplasmic membrane. This process occurs in a rather non-selective way. The isomerization/reduction pathway rearranges improperly introduced disulfides [15,16].

The sequencing of more and more bacterial genomes has revealed that the process of disulfide bond formation in bacteria is extremely diverse, and it has become obvious that *E. coli *Dsb system cannot be considered a paradigm for Dsb activity [16,17]. The Dsb oxidative pathway of *C. jejuni *is much more complex than the oxidative pathway of the laboratory *E. coli *K-12. Depending on the strain, it is catalyzed by three or four enzymes - two localized in the inner membrane (DsbB and DsbI) and one or two in the periplasm (DsbA1 and DsbA2). DsbA1 and DsbA2 possess classic signal sequences, which potentially ensure their transport through the cytoplasmic membrane into the periplasm. They are both directly responsible for disulfide bond formation. DsbB and DsbI, orthologues of *E. coli *DsbB, are potentially involved in DsbA1/DsbA2 re-oxidation [18]. *C. jejuni *genes of the Dsb oxidation pathway are organized in two clusters located at different chromosomal *loci*: *dsbA2*-*dsbB*-*astA*-*dsbA1 *and *dba*-*dsbI*. AstA (arylsulfatase), encoded by the gene located in the first cluster, transfers arylsulfate groups between aromatic substrates in an adenosine 3'-phosphate-5'phosphosulfate (PAPS)-independent manner, at least in an *E. coli *strain [19-21], and is a substrate for the Dsb oxidative pathway. Based on specificity toward the donor aromatic substrate, arylsulfatases are classified as PAPS-dependent or PAPS-independent enzymes. The mode of *C. jejuni *AstA action remains uncharacterized. The *dba *gene encodes a potential protein of unknown function. Except for *dsbA2, C. jejuni dsb *genes are highly conserved within the species. Only *dsbA2 *is variable among strains [15].

An active Dsb system is required for intestinal colonization by *Campylobacter*, as shown in a chicken infection model. Additionally, *C. jejuni *strain 81-176 with a mutated *dsbB *or *dsbI *gene showed reduced invasion/intracellular survival ability in T84 cells. These data indicate that some targets of the Dsb system are involved in crucial processes of *Campylobacter *pathogenicity and commensalism [22].

The goal of this work was to analyze *C. jejuni dsb *oxidative gene expression by characterizing its transcriptional units, and identify control mechanisms and environmental regulatory factors that facilitate the pathogen's adaptation to varying living conditions. We show that the *dsb *genes are arranged in three operons in the genome, and that expression of those operons responds to an environmental stimulus - iron availability. Although transcription of *dsbB *and *dsbI *are both altered by iron concentration with Fur protein engagement, they are regulated differently. Thus, by changing Dsb protein abundance, the pathogen can regulate the amounts of many extracytoplasmic virulence factors that are substrates of the Dsb system, depending on the environmental conditions. Additionally, results show that synthesis of DsbI oxidoreductase is strongly controlled by the mechanism of translational coupling.

## Methods

### Bacterial strains, plasmids, media and growth conditions

Bacterial strains and plasmids used in this study are listed in Table 1. *C. jejuni *strain 81-176 [23], and 480 [24] were grown under microaerobic conditions at 37°C in Mueller Hinton (MH) broth, on MH agar or Blood Agar Base No. 2 (BA) containing 5% horse blood. *E. coli *strains were grown at 37°C in Luria Bertani (LB) broth or on LB agar. When appropriate, the media were supplemented with antibiotics [*Campylobacter *Selective Supplement (Oxoid), ampicillin (100 μg/ml), chloramphenicol (15 μg/ml), kanamycin (30 μg/ml) or tetracycline (10 μg/ml)], iron sulfate Fe_2_(SO_4_)_3 _(40 μM - final concentration in all experimets) or the iron chelator deferoxamine mesylate (20 μM - final concentration in all experimets), X-Gal (13 mg/ml) and/or IPTG (3 mg/ml) in DMF (dimethyl-formamide).

**Table 1:** Bacterial strains and plasmids used in this study

Strain/plasmid	Genotype or relevant characteristics	Origin
***C. jejuni *strains**		
81-176	parental strain; pVir, pTet (Tet^R^)	G. Perez - Perez *
AG1	81-176 *dba*::*aphA-3*	This study
AL1	81-176 *dsbI*::*cat*	This study
AG6	81-176 Δ*dba-dsbI*::*cat*	This study
AG11	81-176 *fur*::*cat*	This study
480	parental strain	J. van Putten **
AL4	480 *dsbI*::*cat*	This study
AG15	480 *fur::cat*	This study
***E. coli*strains**		
DH5α	F^- ^Φ80d *lacZ *ΔM15 Δ(*lacZYA-orgF*)U169 *deoR recA*1*endA*1 *hsdR*17 (r_k_^- ^m_k_^+^) *phoA supE*44 λ^- ^*thi*-1 *gyrA*96 *relA*1	Gibco BRL
TG1	*supE*44 *hsd*Δ 5 *thi *Δ(*lac- proAB*) F' [*traD*36 *proAB *^+ ^*lacI^q ^lacZ*ΔM15]	[26]
S17-1	*recA pro hsdR *RP4-2-Tc::Mu-Km::Tn7 Tmp^r^, Spc^r^, Str^r^	[56]
**General cloning/Plasmid vectors**		
pGEM-T Easy	Ap^r^; LacZα	Promega
pRY107	Km^r^; *E. coli/C. jejuni *shuttle vector	[27]
pRY109	Cm^r^; *E. coli/C. jejuni *shuttle vector	[27]
pRK2013	Km^r^; helper vector for *E. coli/C. jejuni *conjugation	[28]
**Plasmids for gene expression study** Cj stands for PCR-amplified *C. jejuni *81-176 DNA fragment (PCR primers are given in brackets) Cc stands for PCR-amplified *C.coli *72Dz/92 DNA fragment (PCR primers are given in brackets) *cj *stands for *C. jejuni *81-176 gene		
pUWM471	pMW10/1300 bp Cc (H0B - H4X)	[39]
pUWM803	pMW10/440 bp Cj (Cjj879B - Cjj880X)	This study
pUWM792	pMW10/1170 bp Cj (Cjj879B - Cjj881X)	This study
pUWM795	pMW10/1980 bp Cj (Cjj879B - Cjj882X)	This study
pUWM832	pMW10/690 bp Cj (Cjj880B - Cjj880X)	This study
pUWM833	pMW10/750 bp Cj (Cjj880B2 - Cjj881X)	This study
pUWM834	pMW10/900 bp Cj (Cjj881B - Cjj882X)	This study
pUWM864	pMW10/660 bp Cj (Cjj882B3 - Cjj883X2)	This study
pUWM827	pMW10/540 bp Cj (Cj19LX-2 - Cj18Bgl)	This study
pUWM828	pMW10/720 bp Cj (Cj19LX-2 - Cj17Bgl)	This study
pUWM858	pMW10/240 bp Cj (Cjj45B - Cjj44X)	This study
**Plasmids for mutagenesis**		
pAV80	pBluescript II SK/*cjfur::cat*	[25]
pUWM622	pBluescript II KS/*cjdba::aphA-3*	This study
pUWM713	pGEM-T Easy/*cjdsbI::cat*	This study
pUWM867	pGEM-T Easy/Δ*cjdba-cjdsbI::cat*	This study
**Plasmids for translational coupling study**		
pUWM769	pRY107/*cjdba-cjdsbI *operon	This study
pUWM811	pRY107/*cjdba *(M1R)*-cjdsbI *operon	This study
pUWM812	pRY107/*cjdba *(L29stop)*-cjdsbI *operon	This study
pUWM1072	pBluescript II SK/promoter of *cjdba-cjdsbI *operon	This study
pUWM1100	pBluescript II SK/*cjdsbI *with its own promoter	This study
pUWM1103	pRY107/*cjdsbI *with its own promoter	This study
**Plasmid for recombinant protein synthesis and purification**		
pUWM657	pET28a/*cjdsbI *(1100 bp 5'-terminal fragment)	This study
pUWM1098	pET24d/*cjfur *(*fur *coding region)	This study

* New York University School of Medicine, USA

** Utrecht University, The Netherlands.

As previously reported [6], growth of the *C. jejuni *NCTC 11168 was slower in the presence of deferoxamine mesylate (iron-restricted conditions) than in the presence of iron sulfate (iron-rich conditions). This was also observed for *C. jejuni *81-176, so in iron-restricted conditions the strain was cultivated 5-7 hours longer than in iron-sufficient or iron-rich conditions, till the culture reached OD_600 _of about 0.4-0.6. We also noted that growth of the *fur::cat *mutated *C. jejuni *strains was markedly slower in iron-rich conditions than that of the wild type strain, but it was not slower in iron-restricted conditions. A similar inhibitory effect of iron chelation on the growth of *C. jejuni *11168 was previously reported by van Vliet [6,25].

### General DNA procedures

Standard procedures for plasmid DNA isolation and DNA analysis were carried out as described by Sambrook and Russel [26] or were performed according to the manufacturer's instructions (A&A Biotechnology). Synthetic primers synthesis (sequences given in Table 2) and DNA sequencing were performed in the DNA Sequencing and Oligonucleotide Synthesis Laboratory at the Institute of Biochemistry and Biophysics, Polish Academy of Sciences.

**Table 2:** Oligonucleotides used in the present study

Name	Sequence	Orientation//restriction site
H0B	GTCTAGGATCC**GCTTGATATCGCTGATTACT**	**Fwd//BamHI**
H4X	ATCTGTCTAGAGC**CAGCAGGAGCAATTACATCT**	**Rev//XbaI**
Cj16RS	GCAGTCGAC**TCAATGAAGGTA**A**GAGTAAG**	**Rev//SalI**
Cj17Bgl	CCTAGATCT**AGCCTGCTAAACACATTAGT**	**Rev//BglII**
Cj17Nde	GTACAT**ATGAACGAAATCAATAAAAC**	**Fwd//NdeI**
Cj17RBgl	TTCAGATCT**CTAATGTGTTTAGCAGGC**	**Fwd//BglII**
Cj17RM	TATGAATTC**AGGAATACCTGTGCTAACAA**	**Rev//EcoRI**
Cj17LSal	GCTGTCGAC**TGATAAGAAAGAATATTG**	**Rev//SalI**
Cj17WDBam-low	GGATCC**TGTGGGGAGTGCGATAG**	**Rev//BamHI**
Cj17WDBam-up	GGATCC**ACAGGTATTCCTCCTTATGTAG**	**Fwd//BamHI**
Cj18Bgl	CCTAGATCT**GATAATCAGTATCAAGGCGA**	**Rev//BglII**
Cj18L29	**CCAAGCTACCATTACC**t**AACCAAAAGCCAAAT**	**Rev//Ø**
Cj18L29_c	**ATTTGGCTTTTGGTT**a**GGTAATGGTAGCTTGG**	**Fwd//Ø**
Cj18LM	TATGGATCC**CAGGAGCACTATTAACAATA**	**Fwd//BamHI**
Cj18M1R	**AAAGTTCAAGAAACTCC**c**TAGTATCTCCTTTG**	**Rev//Ø**
Cj18M1Rc	**CAAAGGAGATACTA**g**GGAGTTTCTTGAACTTT**	**Fwd//Ø**
Cj18Nde-Rev	**ATA**CTGCAG**CAAGAAACTC**CATATG**ATCTCC**	**Rev//PstI, NdeI**
Cj18RM	TGAGGATCC**AAGCCAAGCTACCATTACCA**	**Rev//BamHI**
Cj19LX-2	AGTTCTAGA**AGTTGGACAGCTTGCTGATA**	**Fwd//XbaI**
Cjj43Eco	GCAGAATTC**AAGCATAGCAGGATCTTTGG**	**Rev//EcoRI**
Cjj43mwL	CG***TGGATCCCCGGGTA*GGACTTATATTTAATC**	**Fwd//SmaI, BamHI**
Cjj44X	AGTTCTAGA**CATTTAGCCCTTCCTTACAG**	**Rev//XbaI**
Cjj45B	ATCGGATCC**GATACTATGGAGTTTCTTGA**	**Fwd//BamHI**
Cjj45Dig	**TCAAGAAACTCCATAGTATC**	**Rev//Ø**
Cjj46	**CATGTGAAATCAATAATATC**	**Fwd//Ø**
Cjj46mwR	A***TACCCGGGGATCCA*AAGTTACTGAAAGCTAC**	**Rev//SmaI, BamHI**
Cj-RT	GCAGTCGAC**T**A**GGATCGATAG**T**AGCTGAA**	**Rev//SalI**
Cjj879B	ATCGGATCC**CACAATCTAAAGGGTATTTC**	**Fwd//BamHI**
Cjj880	**CTTATCCATAAAATATAATG**	**Fwd//Ø**
Cjj880B	ATCGGATCC**ACCTAGATTATTCTACTTTG**	**Fwd//BamHI**
Cjj880B2	ATCGGATCC**TTTCCAGTAAAATTAGCAAG**	**Fwd//BamHI**
Cjj880X	AGTTCTAGA**CACATACAACAATAGATCTT**	**Rev//XbaI**
Cjj881B	ATCGGATCC**AAAATGAAAGATAATTGCAG**	**Fwd//BamHI**
Cjj881X	AGTTCTAGA**AAATGTGCTATACAAGTAAG**	**Rev//XbaI**
Cjj882	**AGGTGTAAGTCTTGGAGAGC**	**Fwd//Ø**
Cjj882B	ATGGATCC**GACTTAGCAAAACTCTTTGT**	**Fwd//BamHI**
Cjj882X	AGTTCTAGA**AGAGTTTTGCTAAGTCTCAT**	**Rev//XbaI**
Cjj882B3	ATCGGATCC**ATGATGATTATAGCAAATGC**	**Fwd//BamHI**
Cjj883X2	AGTTCTAGA**GCTAATGCAAAACTTGAATA**	**Rev//XbaI**
CM-L	**ATATCACGCAATTAACTTGG**	**Rev//Ø**
CM-R	**GGATGAATTACAAGACTTGC**	**Fwd//Ø**
DIG_dsbA1	**GCTAATGCAAAACTTGAATA**	**Rev//Ø**
DIG_dsbA2X	**CACATACAACAATAGATCTTG**	**Rev//Ø**
DIG_chuF	**CATATGAGAAATAATGCTTTC**	**Fwd//Ø**
EMSAchuR	**TTTGGGTGCAAATTTTACTC**	**Rev//Ø**
Fur-L	**TGAATTTTTATTGGTTTGATGC**	**Fwd//Ø**
Fur-R	**TCCTCATCTTCAATGTTTGC**	**Rev//Ø**
KAN-L	**TATCACCTCAAATGGTTCGCTGGG**	**Rev//Ø**
KAN-R	**GGGGATCAAGCCTGATTGGGAGA**	**Fwd//Ø**
KM-L1	**GAGAATATCACCGGAATTGA**	**Fwd//Ø**
KM-R1	**CTTCATACTCTTCCGAGCAA**	**Rev//Ø**
lacZ	AGGTTACGTTGGTGTAGATG	**Rev//Ø**
lacZ1	GGAATTCACTGGCCGTCGTT	**Fwd//Ø**

**Bold letters **indicate *C. jejuni *81-176 sequences; restriction recognition sites introduced for cloning purposes are underlined, complementary fragments of primers Cjj46mwR and Cjj43mwL are marked with *italics*. Point mutated nucleotides in primers are marked with **small letters**. Orientation of the primers (**Fwd **states for forward/**Rev **- for reverse) refers to the orientation of particular *C. jejuni *gene studied. RT-Cj primer was designed on the basis of *C. coli *72Dz/92 *dsbI *nucleotide sequence (there are 2 nucleotide changes compared to the nucleotide sequence of its orthologue from *C. jejuni *81-176).

All vectors containing transcriptional fusions of putative *dsb *gene promoter regions with a promotorless *lacZ *gene were constructed using the pMW10 *E. coli/C. jejuni *shuttle vector. DNA fragments were amplified from *C. jejuni *81-176 chromosomal DNA with appropriate pairs of primers (listed in Table 2). Next, PCR products were cloned in the pGEM-T Easy vector (Promega), excised by restriction enzymes and subsequently cloned into pMW10, forming transcriptional fusions with the downstream promoterless *lacZ *reporter gene. Correct construction of the resulting shuttle plasmids was confirmed by restriction analysis and sequencing. All recombinant plasmids, as well as the empty pMW10, were introduced into *C. jejuni *480 cells by electroporation.

Construction of a pUWM1072 plasmid containing *dsbI *without *dba *under its native promoter was achieved by PCR-amplification of the 520 bp chromosomal DNA fragments containing the *dba-dsbI *promoter sequences (primer pair Cj19LX-2 - Cj18Nde-Rev) and cloning it into pBluescript II SK (Stratagene), using XbaI/PstI restriction enzymes. Subsequently the *dsbI *coding sequence (1792 bp) was PCR-amplified using the Cj17Nde - Cj16RS primer pair, cloned into pGEM-T Easy (Promega) and finally, using NdeI/SalI restriction enzymes, transferred into pUWM1072 in the native orientation, generating the plasmid pUWM1100. The whole insert (2316 bp) was then cloned into a shuttle *E. coli/C. jejuni *vector pRY107 [27] using SalI/XbaI restriction enzymes. The resulting, plasmid pUWM1103, whose correct construction was verified by sequencing, was used for complementation assays in *C. jejuni *Δ*dba-dsbI::cat *mutant cells.

Point mutations were generated using a Quick-Change site-directed mutagenesis kit, following the supplier's recommendations (Stratagene). To construct a *dba *gene with point mutations, the pUWM456 plasmid, containing the *C. jejuni dba-dsbI *genes, was used as a template for PCR-mediated mutagenesis. Point mutations M1R and L29stop (replacing a Leu codon with amber stop codon) were introduced using the respective pairs of primers: Cj18M1R - Cj18M1Rc and Cj18L29 - Cj18L29c. The resulting plasmids were introduced into *E. coli *cells by transformation and presence of desired mutations was verified by DNA sequencing. DNA fragments containing the *C. jejuni dba*-*dsbI *operon (with or without a point mutation) were then digested and inserted into the pRY107 shuttle vector. The resulting plasmids were named pUWM769 (containing wt *dba-dsbI*), pUWM811 (*dba*: M1R, wt *dsbI*) and pUWM812 (*dba*: L29stop, wt *dsbI*). These plasmids were subsequently introduced into *C. jejuni *81-176 AL1 (*dsbI::cat*) and *C. jejuni *81-176 AG6 (Δ*dba-dsbI::cat*) knock-out cells by conjugation [28].

### Construction of bacterial mutant strains

To inactivate *dba *and *dsbI *genes, three recombinant plasmids were constructed, based on pBluescript II KS (Stratagene) and pGEM-T Easy (Promega) vectors, which are suicide plasmids in *C. jejuni *cells. A. van Vliet kindly furnished the fourth suicide plasmid, pAV80, which was previously used for *C. jejuni *NCTC11168 *fur *inactivation [25]. Correct construction of all the plasmids was confirmed by restriction analysis and sequencing.

The plasmid for *C. jejuni dba *mutagenesis was generated by PCR-amplification of two *C. jejuni *81-176 DNA fragments (600 bp and 580 bp long) that contained *dba *gene fragments with their adjacent regions with primer pairs: Cj19LX-2 - Cj18RM and Cj18LM - Cj17RM. Next they were cloned in native orientation in pBluescript II KS (Statagene). Using BamHI restrictase, the kanamycin resistance cassette (the 1.4 kb *aphA-3 *gene excised from pBF14) was inserted between the cloned *dba *arms in the same transcriptional orientation, generating the suicide plasmid pUWM622.

To obtain the construct for *C. jejuni dsbI *mutagenesis the 1.5 kb DNA fragment containing the *dsbI *gene was PCR-amplified from the *C. jejuni *81-176 chromosome using primer pair: Cj17LSal - Cj17RBgl and was cloned into pGEM-T Easy (Promega). Subsequently, the internal 300 bp EcoRV-EcoRV region of *dsbI *was replaced by a SmaI-digested chloramphenicol resistance cassette (the 0.8 kb *cat *gene excised from pRY109) [27] inserted in the same transcriptional orientation as the *dsbI *gene, generating the suicide plasmid pUWM713.

To obtain the construct for *C. jejuni dba-dsbI *mutagenesis, the 410 bp and 380 bp DNA fragments, containing *dba *upstream and *dsbI *downstream regions were PCR-amplified from the *C. jejuni *81-176 chromosome using primer pairs: Cj19LX-2 - Cjj46mwR and Cjj43mwL - Cjj43Eco. These fragments were directly digested with BamHI restrictase, ligated in a native orientation and used as a template for a subsequent PCR reaction with the external primer pair: Cj19LX-2 - Cjj43Eco. This PCR product was cloned into pGEM-T Easy (Promega) and the chloramphenicol resistance cassette (the 0.8 kb *cat *gene excised from pRY109) was inserted in the same transcriptional orientation as *dba-dsbI *operon at the BamHI site between the *C. jejuni *DNA fragments, generating suicide plasmid pUWM866.

Gene versions inactivated by insertion of a resistance cassette were introduced into the *C. jejuni *81-176 or 480 chromosome by the allele exchange method as described by Wassenaar *et al*. [24]. Construction of the *C. jejuni *480 *fur::cat *mutant was achieved by natural transformation using *C. jejuni *81-176 *fur::cat *chromosomal DNA. It should be pointed out that *C. jejuni *480 was previously described as incapable of accepting chromosomal DNA by natural transformation [24]. Such inconsistency of experimental data might be due to different chromosomal DNA used for natural transformation (*C. jejuni *81116 *vs C. jejuni *81-176). The mutant strains were obtained by two- or tri-parental mating experiments performed as described by Labigne-Roussel *et al*. [29] and Davis *et al*. [30]. The constructed mutants were named AG1 (*C. jejuni *81-176 *dba::aphA-3*), AL1 (*C. jejuni *81-176 *dsbI::cat*), AL4 (*C. jejuni *480 *dsbI::cat*), AG6 (*C. jejuni *81-176 Δ*dba-dsbI::cat*), AG11 (*C. jejuni *81-176 *fur::cat*), and AG15 (*C. jejuni *480 *fur::cat)*. They demonstrated normal colony morphology and all but two had normal growth rates when cultured on BA plates. Only the *C. jejuni *81-176 *fur::cat *and *C. jejuni *480 *fur::cat *exhibited slower growth, an observation consistent with other studies on *fur *mutants [25]. Disruption of each gene as a result of double cross-over recombination was verified by PCR with appropriate pairs of primers flanking the insertion site (Table 2). The loss of DsbI synthesis in the constructed mutants was verified by Western blotting of whole-cell protein extracts against specific rabbit polyclonal anti-rDsbI antibodies.

### Protein manipulation, and β-galactosidase and arylsulfate sulfotransferase (AstA) assays

Preparation of *C. jejuni *protein extracts, SDS-PAGE (sodium dodecyl sulfate polyacrylamide gel electrophoresis) and blotting procedures were performed by standard techniques [26].

To obtain recombinant His_6_-DsbI protein, the 1100 bp DNA fragment containing the coding sequence for the predicted periplasmic DsbI C-region was PCR-amplified from the *C. jejuni *81-176 chromosome using a primer pair: Cj17WDBam-up - Cj17WDBam-low. This fragment was cloned into the pGEM-T Easy vector and then, using BamHI restriction enzyme, into expression vector pET28a (Novagen) to generate plasmid pUWM657, whose correct construction was verified by restriction analysis and sequencing. Cytoplasm-located soluble fusion protein His_6_-DsbI purified from the *E. coli *Rosetta (DE3) LacI^q ^strain by affinity chromatography was used for rabbit immunization (Institute of Experimental and Clinical Medicine, Polish Academy of Science, Warsaw, Poland). The anti-His_6_-DsbI (anti-rDsbI) serum obtained was highly specific and recognized native DsbI, as verified by Western blot experiments carried out with protein extracts from *C. jejuni *wild type and a *dsbI *mutant strain (data not shown).

To obtain recombinant Fur-His_6 _protein, the DNA fragment containing the entire *fur *coding region was PCR-amplified from the *C. jejuni *81-176 chromosome with primer pair CjFurNcI - CjFurXhI, and then cloned, using NcoI/XhoI restriction enzymes, into pET24d (Novagen). This generated pUWM1098, carrying a *fur-his_6 _*translational fusion. This plasmid was then transformed into *E. coli *BL21 (DE3) cells. Recombinant Fur-His_6 _protein was overproduced by addition of 1mM IPTG to the bacterial culture at exponential growth phase and purified under native conditions by affinity chromatography.

β-galactosidase activity assays in *C. jejuni *cell extracts were performed three times (each time three independent samples were taken for each strain), as described by Miller [31].

*C. jejuni *transformants grown overnight on BA medium were harvested and resuspended in LB medium to achieve comparable cell densities (OD_600 _approx. 0.6). Fresh MH liquid medium (MH supplemented with iron sulfate - iron-rich conditions, MH itself - iron-sufficient and MH with iron chelated by addition of deferoxamine mesylate - iron-restricted conditions) was inoculated with *C. jejuni *(1:10) and incubated overnight (15-22 h depending on the medium) till the culture reached OD_600 _of about 0.4-0.6. Since Wright *et al*. documented that *C jejuni *exhibits a dynamic stationary phase, characterized by switches in motility, substrate utilization and metabolite production accompanied by concurrent changes in gene expression, exponential phase cultures were used in this experiment to eliminate any stationary phase-dependent physiological switching of gene expression levels [32].

Quantitative assays for AstA arylsulfatase activity were performed three times (each time three independent samples were taken for each strain), using the method described by Hendrixson *et al*. with one difference: the *C. jejuni *81-176 strain was cultivated on MH liquid medium under high- or low-iron conditions [33] (approx. 17 h on MH medium under high iron condition and approx. 22 h on MH medium under low-iron condition). For each experiment, bacterial cultures of the same OD (OD_600 _~ 0.6-0.7) were used.

### RNA analysis

Total RNAs were extracted from *C. jejuni *overnight BA culture using the standard TRIzol reagent according to the manufacturer's protocol (Invitrogen). RNA samples were treated with DNaseI to eliminate contaminating DNA and quantified by measurements of OD_260_, RNA was reverse transcribed using Superscript II enzyme (Invitrogen) and RT-primer (Table 2): Cj-RT complementary to the *dsbI*-internal fragment, or KM-R1, complementary to the kanamycin-resistance cassette. The RT primer was annealed stepwise before adding the reverse transcriptase. The enzyme was finally inactivated by incubation at 70°C for 15 min. A control reaction without reverse transcriptase was used to determine RNA template purity from DNA. PCR reactions (with pairs of primers: Cj17Nde - Cj17RM or KM-L1 - KM-R1) performed on cDNA were carried out in the presence of 2 mM MgCl_2 _using the following protocol: initial denaturation at 94°C for 5 min; then 30 cycles of: 30 s denaturation at 94°C, 30 s annealing at 50-60°C, 30-180 s elongation at 72°C and 10 min terminal elongation at 72°C. Resulting PCR products were separated by electrophoresis in a 1.5% agarose gel.

RNA secondary structure was predicted by calculating a 100% consensus among different methods (Afold, PknotsRG, RNAfold, Contrafold, and RNAsubopt) run *via *the metaserver available at http://genesilico.pl/rnametaserver/.

### Gel mobility shift assay

The promoter regions upstream of the *dba-dsbI *and *dsbA2-dsbB-astA *operons (~180 bp and ~330 bp, respectively) and the *dsbA1 *gene (~300 bp) as well as the *CJJ81176_1600 *- *chuA *intergenic spacer region (~220 bp) which contains two Fur boxes (positive control) were PCR-amplified from *C. jejuni *81-176 chromosomal DNA, using the following primer pairs: DIG_Cjj45 - Cjj46, DIG_dsbA2X - Cjj880, DIG_dsbA1 - Cjj882 and DIG_chuF - EMSAchuR. Primers: DIG_Cjj45, DIG_dsbA2X, DIG_dsbA1 and DIG_chuF were digoxigenin labelled (Metabion). Approximately 28 fmol of each DIG-labelled DNA fragment was incubated with 0, 333, 1000 or 3333 nM of purified Fur-His protein for 20 min. at room temperature and subsequently for 5 min. at 37°C in a 20 μl volume of binding buffer routinely used for the Fur-binding assay (10 mM Tris-HCl [pH 7.5], 1 mM MgCl_2 _,0.5 mM dithiothreitol, 50 mM KCl, 100 μM MnCl_2_, 1 μg poly (dI-dC), 50 μg bovine serum albumin and 5% glycerol). In addition, *dsbA2 *and *dsbA1 *promoter regions were incubated with Fur-His protein in binding buffer without Mn^2+^. As negative controls each Dig-labelled DNA fragment was incubated with an unrelated protein (purified *H. pylori *HP0377- His_6_). Control reactions were performed using competitor DNA - unlabeled promoter DNA region. Samples were run on a 5% non-denaturing Tris-glycine polyacrylamide gel at 4°C. Then DNA was transferred to nylon membranes (Roche) and UV cross-linked. Labelled DNA was detected with anti-DIG antibody using a standard DIG detection protocol (Roche).

## Results

### *In silico *analysis of *C. jejuni *81-176 *dsb *gene clusters

**Figure 1:**
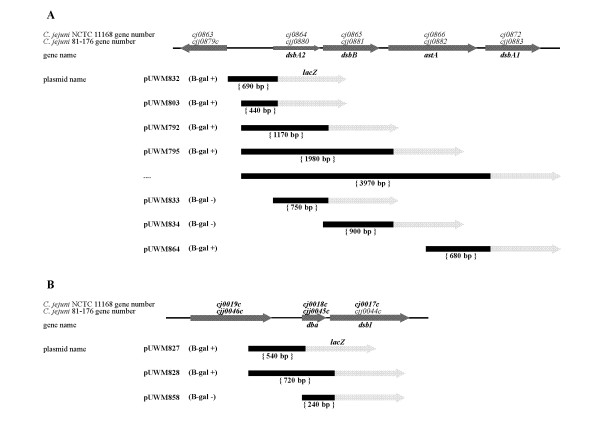
**Organization of *dsb *genes in the *C. jejuni *81-76 chromosome and constructs prepared for *dsb *transcription studies; the *dsbA2-dsbB-astA-dsbA1 *gene set (A), the *dba-dsbI *gene set (B)**. Hazy grey boxes stand for *C. jejuni *genes (*C. jejuni *NCTC 11168 and 81-176 gene numbering is given above the boxes, below them the studied gene names are given). Black boxes stand for the *C. jejuni *81-176 DNA fragments cloned in the transcriptional fusions with the promoterless *lacZ *gene, displayed by the light grey boxes. The longest transcriptional fusion could not be obtained. Sign **β-gal +/- **at the right side of the plasmid name stands for presence/absence of β-galactosidase activity conferred by the appropriate construct for the transformant cells.

*C. jejuni *81-176 *dsbA2-dsbB-astA-dsbA1 *genes (*cjj81176_0880-0883*) have the same orientation in the chromosome (Figure 1A) and are separated by short intergenic regions - 11 bp, 87 bp, and 85 bp, respectively. Thus, they potentially might be co-transcribed. *In silico *analysis of the *C. jejuni dsbA2-dsbB-astA-dsbA1 *cluster revealed the presence of a potential RBS as well as a complete promoter nucleotide sequence upstream of *dsbA2*, located within the 627 bp intergenic *xerD*-*dsbA2 *region [34]. As this DNA fragment consists of -35, -16 and -10 regions (characteristic for the σ^70^ binding sequence), it can be recognized by *Campylobacter *RNAP containing the main sigma factor. Directly upstream of *dsbB *there is a potential additional RBS sequence but none of the promoter regions were found, suggesting *dsbA2-dsbB *co-transcription. Upstream of *astA *and *dsbA1 *there are putative RBS sequences and incomplete promoter nucleotide sequences, suggesting that *astA *and *dsbA1 *might be transcribed separately from *dsbA2 *and *dsbB*.

*C. jejuni *81-176 *dba *(*cjj81176_0045c*) and *dsbI *(*cjj81176_0044c*) have the same orientation in the chromosome (Figure 1B) and their coding sequences are separated by a short intergenic region of 11 bp. An initial RT-PCR experiment carried out on the total *C. jejuni *RNA documented *dba-dsbI *co-transcription *in vitro *and localization of their promoter within 493 bp DNA upstream of the *dba *translation start codon [18].

### Transcriptional analysis of two *dsb *gene clusters

The *lacZ *reporter gene system was used to determine the *dsb *gene expression and regulation. Two sets of *dsb-lacZ *transcriptional fusions were designed based on a promotorless *lacZ *gene in the shuttle vector pMW10 [34]. The first one comprised of seven plasmids (pUWM792, pUWM795, pUWM803, pUWM832, pUWM833, pUWM834 and pUWM864) employed to study *dsbA2*/*dsbB/astA/dsbA1 *expression. The other consisted of three plasmids (pUMM827, pUWM828 and pUWM858) generated to analyze *dba*/*dsbI *expression. Details of the recombinant plasmid structures are shown in Figure 1. We successfully prepared all but one of the planned transcriptional fusions - we failed at constructing the longest fusion presented in Figure 1.

β-galactosidase assays indicated that the fusions present in pUWM833, pUWM834 and pUWM858 were not expressed in *C. jejuni *cells. This documented that the analyzed genes form two polycistronic operons (*dsbA2-dsbB*-*astA *and *dba-dsbI*) and only *dsbA1 *is independently transcribed. The level of β-galactosidase provided by the *dsbA1 *promoter was approximately ten times higher than that conferred by the two other promoters that were analyzed (contained in pUWM803 and pUWM827). Thus, three promoters of various strengths and responsible for *C. jejuni dsb *gene expression were identified: P*_dbadsbI_*, P*_dsbA2dsbBastA _*and P*_dsbA1_*.

### Influence of environmental stimuli on *dsb *gene expression

**Figure 2:**
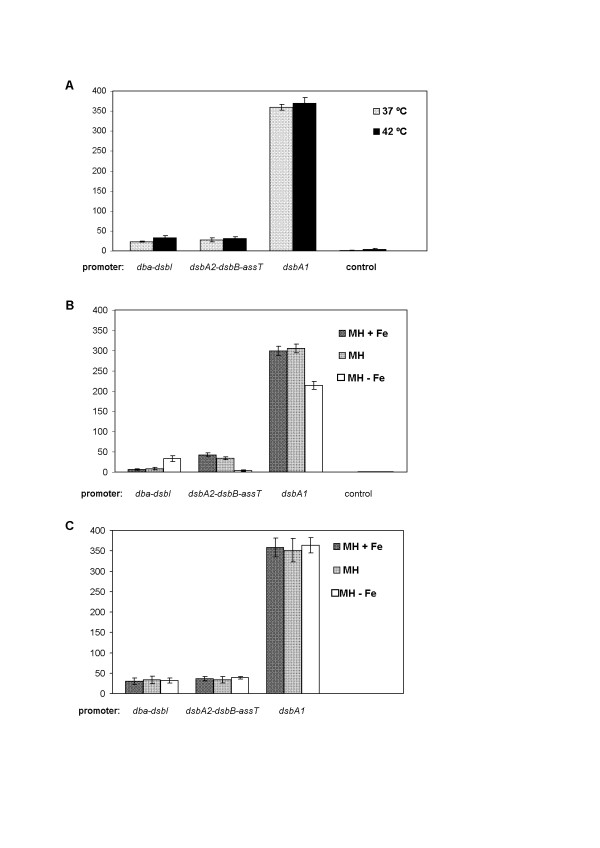
**Transcription levels of *C. jejuni *81-76 *dsb *genes (measured by β-galactosidase activity assays) in the wild type strain (A and B) and *fur::cat *mutant (C) under different environmental conditions**. Each experiment was repeated three times, and each time three independent samples were taken for each strain (giving 9 independent measurements for each strain). Statistical significance was calculated using t-Student test for comparison of independent groups (GraphPad Prism). The wild type strain *C. jejuni *480 carrying an empty vector pMW10 was used as a control. Statistical p values: For wild type *C. jejuni *480 strain: P*_dba-dsbI _*temp. 37°C *vs *42°C: p = 0,0001(*). P*_dsbA2-dsbB-astA _*temp. 37°C *vs *42°C: p = 0,2020. P*_dsbA1 _*temp. 37°C *vs *42°C: p = 0,1031. P*_dba-dsbI _*MH+Fe *vs *MH: p = 0,0576. P*_dba-dsbI _*MH-Fe *vs *MH: p < 0,0001(*). P*_dsbA1-dsbB-astA _*MH+Fe *vs *MH: p = 0,0007(*). P*_dsbA1-dsbB-astA _*MH-Fe *vs *MH: p < 0,0001(*). P*_dsbA1 _*MH+Fe *vs *MH: p = 0,2569. P*_dsbA1 _*MH-Fe *vs *MH: p < 0,0001(*). For mutant *C. jejuni *480 *fur::cat *strain: P*_dba-dsbI _*MH+Fe *vs *MH: p = 0,3683. P*_dba-dsbI _*MH-Fe *vs *MH: p = 0,6796. P*_dsbA1-dsbB-astA _*MH+Fe *vs *MH: p = 0,3164. P*_dsbA1-dsbB-astA _*MH-Fe *vs *MH: p = 0,0577. P*_dsbA1 _*MH+Fe *vs *MH: p = 0,5228. P*_dsbA1 _*MH-Fe *vs *MH: p = 0,2388. P values of P < 0.05 were considered to be statistically significant; they are marked with (*).

We subsequently tested whether gene expression driven by P*_dsbA2dsbBastA_*, P*_dsbA1 _*and P*_dbadsbI _*(*C. jejuni *480 strains harbouring pUWM803, pUWM864 or pUWM827) responds to environmental stimuli. While there were no significant differences in β-galactosidase activity between cells grown at various temperatures (37°C and 42°C) (Figure 2A) or between cells grown in solid and liquid medium (MH broth and MH solidified by agar addition) (data not shown), transcription from each of the analyzed promoters was iron-regulated (Figure 2B). For cells grown in iron-restricted conditions, P*_dsbA2dsbBastA _*activity was 10 times lower, P*_dsbA1 _*activity was about 30% lower, and P*_dbadsbI _*activity was four times higher, compared to cells grown under iron-sufficient/iron-rich conditions.

Iron-regulated expression of many Gram-negative bacterial genes is mediated by the ferric uptake regulator (Fur) [35,36]. Classically, the Fur protein first binds to its co-repressor Fe^2+ ^, and then binds to the conserved DNA sequence (Fur-box) of the regulated promoter, repressing its transcription. However, transcriptomic analyses documented that apo-Fur (without complexed co-repressor) can also influence gene transcription in response to iron concentration [6,36-38].

We therefore decided to evaluate the regulatory function of the Fur protein on *dsb *gene expression. For this purpose a *C. jejuni *480 *fur *isogenic mutant was constructed. Then, recombinant plasmids containing *dsb *promoter-*lacZ *fussions (pUWM803, pUWM864 and pUWM827) were introduced into the *C. jejuni *480 *fur::cat *mutant by electroporation. The results of β-galactosidase assays performed on the constructed strains proved Fur involvement in iron-dependent regulation of the three analyzed *dsb *gene promoters (Figure 2C). β-galactosidase activity conferred by the pUWM827 fusion increased under iron-sufficient/rich conditions in the *fur *mutant as compared to the wild-type strain, suggesting that inactivation of *fur *results in derepression of P*_dbadsbI_*. In contrast, β-galactosidase activities of the pUWM803 and pUWM864 fusions increased under iron starvation in the *fur *mutant compared to the wild-type strain. This indicates that low level of iron leads to Fur-mediated repression of the P*_dsbA2dsbBastA _*and P*_dsbA1 _*promoters, since repression was abolished in the *fur *mutated strain. *C. jejuni *480 strain containing pUWM471, which harbors *cjaA *gene promoter fused to a promotorless *lacZ *gene, was employed as a control in all experiments analyzing the influence of Fur and iron on *dsb *gene expression. There were no significant differences in β-galactosidase activity between wild type cells harbouring pUWM471 grown at various iron concentrations as well as between wt and *fur *mutated cells containing pUWM471. In every case high β-galactosidase levels (about 2000 Miller units) were observed, which is consistent with previously published data that ranked the *cjaA *promoter as one of the the strongest *Campylobacter *spp. promoters so far described [39].

**Figure 3:**
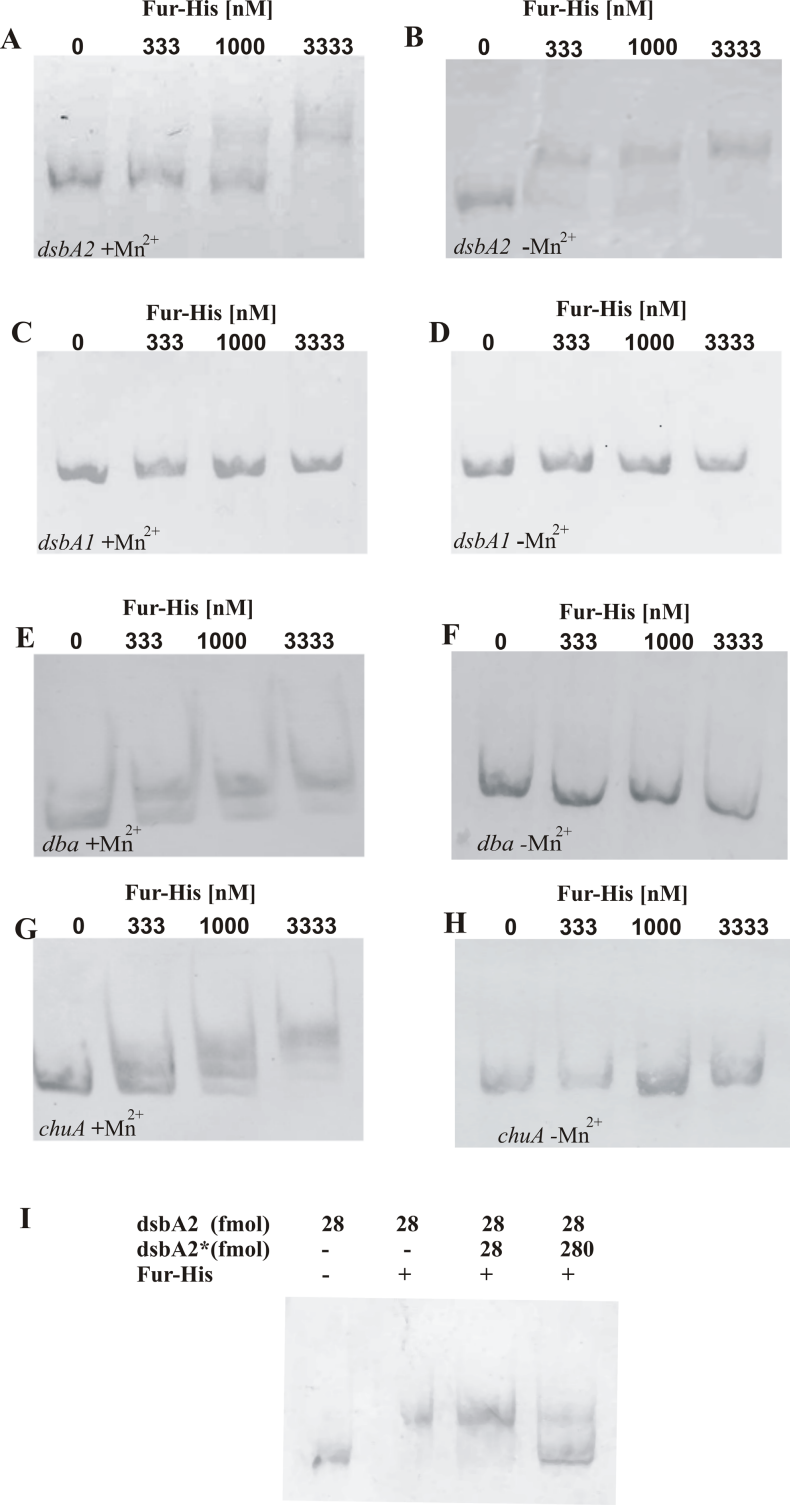
**Electrophoretic mobility shift assays of *chuA, dba-dsbI, dsbA2 *and *dsbA1 *promoter regions bound by CjFur-His_6_**. 28 fmol of Dig-labelled PCR amplified DNA fragments: *dsbA2 *(333 bp - panel A and B), *dsbA1 *(299 bp- panel C and D), *dba-dsbI *(174 bp - panel E and F) and *chuA *(216 bp- panel G and H) were incubated with 0, 333, 1000 or 3333 nM of purified Fur protein. The concentration of CjFur-His_6 _used in the reactions is indicated above the lanes. Binding buffer used in four EMSA studies (panels B, D, F, H) does not contain Mn^2+^. Panel I presents competition gel mobility shift assay which was performed by incubation of 3333 nmol Fur-His protein with 28 fmol of the labelled promoter region upstream of *dsbA2-dsbB-astA *operon (*dsbA2*) and various concentrations of the unlabelled promoter region upstream of *dsbA2-dsbB-astA *operon (dsbA2*)

Inspection of the nucleotide sequences located upstream of the *dba *translation initiation codon did not reveal the presence of an exact *C. jejuni *Fur-binding site sequence motif [40]. So far, a potential Fur binding site for promoters positively regulated by iron concentration in a Fur- dependent manner has not been determined. Therefore, we used EMSA to gain insight into the mechanism by which P*_dbadsbI_*, P*_dsbA2dsbBastA _*and P*_dsbA1 _*are regulated by Fur. To achieve this goal, various primers were designed to amplify a 174 - 299 bp DNA fragment upstream from the translational start site of each tested operon. The promoter region of the *chuA *gene, which contains the Fur-binding motif and is strongly repressed by iron-complexed Fur, was used as a control [6,40]. Mn^2+ ^ions were used in the EMSA in place of Fe^2+ ^due to their greater redox stability. It was demonstrated that the Fur-His_6 _was able to bind *in vitro *to the DNA region upstream of the *dba-dsbI *operon only when the regulatory protein was complexed with Mn^2+^, which indicated, in accordance with previously presented data, that this operon is repressed by the iron-complexed form of Fur (Figure 3E). This promoter region interacts with Fur complexed with Mn^2+ ^as much as the *chuA *promoter (Figure 3G). In contrast, the upstream DNA region of the *dsbA1 *gene did not bind Fur, regardless of the presence of Mn^2+ ^in the reaction buffer. This suggested an indirect method of regulation (Figure 3, panel C and D). In the case of the *dsbA2-dsbB-astA *promoter region, Fur protein bound DNA in the absence of Mn^2+ ^acted as a repressor (Figure 3B), supporting the results obtained in the β-galactosidase assays. Fur-His_6 _complexed with Mn^2+ ^was also able to bind to this DNA fragment but only under conditions of high protein concentration. The formation of DNA/Fur complexes specific for the *dsbA2-dsbB-astA *promoter region was efficiently inhibited by adding unlabelled DNA containing the same DNA fragment.

To check whether the abundance/activity of Dsb-dependent proteins is conditioned by iron concentration, we compared the arylsulfate sulfotransferase (AstA) activity in *C. jejuni *81-176 wt cells grown under iron-restricted to iron-sufficient/iron-rich conditions. As mentioned before, arylsulfatase is a periplasmic direct substrate of the Dsb oxidative pathway [41-43]. This experiment confirmed the dependence of AstA activity on iron concentration. AstA activity of *C. jejuni *81-176 wt grown under iron-restricted conditions reached 75-80% of activity observed for the same strain grown under iron-rich condition (Additional file 1).

### *C. jejuni dba-dsbI *translational coupling

Previously performed *in vitro *transcription/translation coupled assays suggested that *C. jejuni *Dba may influence DsbI synthesis and/or stability [18]. To reveal details of *dba-dsbI *operon expression we examined whether *dba*/Dba was required for *in vivo *synthesis of DsbI in *E. coli *cells. It was demonstrated that in *E. coli*, DsbI underwent partial degradation (for details see Additional file 2 and 3). This result was in agreement with those derived from previous *in vitro *experiments. It is noteworthy that in *C. jejuni *cells, DsbI is produced in two forms as a result of posttranslational modification by glycan binding (for details see Additional file 2 and 4).

**Figure 4:**
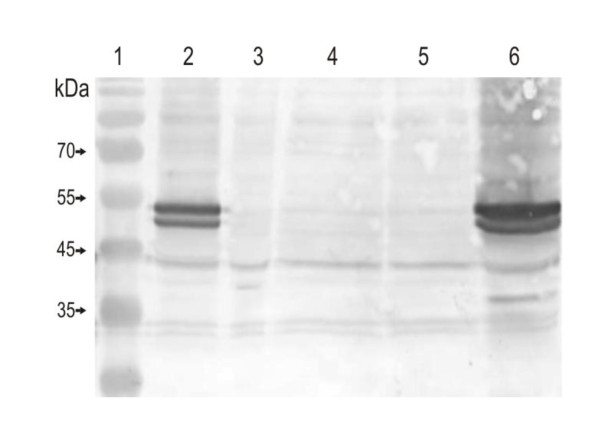
**Translational coupling of *C. jejuni dba*-*dsbI***. Western blot (anti-rDsbI) analysis of *C. jejuni *protein extracts separated by 12% SDS-PAGE. Relative positions of molecular weight markers (lane 1) are listed on the left (in kilodaltons). Lanes 2-6 contain 15 μg of total proteins from: *C. jejuni *81-176 wt (2), *C. jejuni *81-176 AG6 (*dba-dsbI::cat*) (3), AG6/pUWM811 (4), AG6/pUWM812 (5) and AG6/pUWM769 (6)

**Figure 5:**
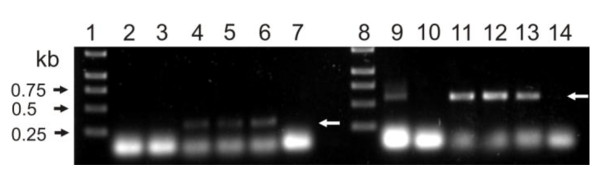
**Analysis of *C. jejuni dsbI *transcription from a *dba-dsbI *operon containing wild type or point mutated *dba***. RT-PCR analysis of *dsbI *(and *aphA-3*) transcription in *C. jejuni *wild type and mutant cells. Equal amounts of mRNAs isolated from *C. jejuni *cells were reverse-transcribed using primer KM-R1 or Cj-RT and resulting cDNA was PCR-amplified with primer pairs KM-L1 - KM-R1 (lanes 1-7) or CjNde - Cj17RM (lanes 8-14), respectively. Relative positions of DNA molecular length markers (lanes 1, 8) are listed on the left (in base pairs). Lanes 2-6 and 9-13 contain PCR products amplified on cDNAs for *C. jejuni *81-176 wt (2, 9), AG6 (*dba-dsbI::cat*) (3, 10), AG6/pUWM811 (4, 11), AG6/pUWM812 (5, 12), AG6/pUWM769 (6, 13); lanes 7 and 14 contain PCR products amplified on RNA for AG6/pUWM769 (after DNase treatment). White arrows indicate products of expected size.

Additionally a *C. jejuni *81-176 isogenic *dba *mutant was constructed by inserting the kanamycin resistance cassette in the same orientation as *dba *coding sequence. This insertion should not alter the downstream *dsbI *transcription. Nevertheless, inactivation of *C. jejuni dba *resulted in the absence of DsbI, and subsequent RT-PCR experiments, conducted for four independently isolated transformants, also documented the absence of *dsbI *transcript in *dba *mutated cells (data not shown). To further examine the role of *dba *expression in DsbI synthesis, a double mutant strain - *C. jejuni *Δ*dba-dsbI::cat *(AG6) - was constructed. Thereafter three recombinant shuttle plasmids, pUWM769 (containing the wild type *C. jejuni dba-dsbI *operon), pUWM811 and pUWM812 (containing point mutated *dba *- M1R or *dba*: L29stop, respectively, and wild type *dsbI*) were introduced into mutant cells. Transformant cells were screened for DsbI synthesis by Western blot analysis with specific rabbit anti-rDsbI serum and additionally by RT-PCR for the presence of *dsbI *transcript. Introduction of pUWM769 into *C. jejuni *81-176 AG6 (Δ*dba-dsbI::cat*), cells resulted in restoration of DsbI production in a higher amount compared to the wild type strain (Figure 4, lane 6), due to plasmid-encoded *dba-dsbI *gene expression. When *dba *translation was completely aborted (*C. jejuni *AG6 carrying pUWM811) and when the truncated 28 aa Dba was produced (*C. jejuni *AG6/pUWM812), DsbI was not synthesized at all (Figure 4, lane 4 and 5, respectively). RT-PCR experiments proved that point mutations in *dba *did not influence *dsbI *transcription, as comparable amounts of *dsbI *mRNA were detected in all but one (AG6) of the strains (Figure 5, lanes: 9, 11-13). Comparable results were obtained for series of *C. jejuni dsbI*::*cat *strains carrying pUWM769, pUWM811 and pUWM812 plasmids (data not shown), suggesting that intact, chromosomally-encoded Dba cannot act *in-trans *to ensure *dsbI *mRNA translation.

**Figure 6:**
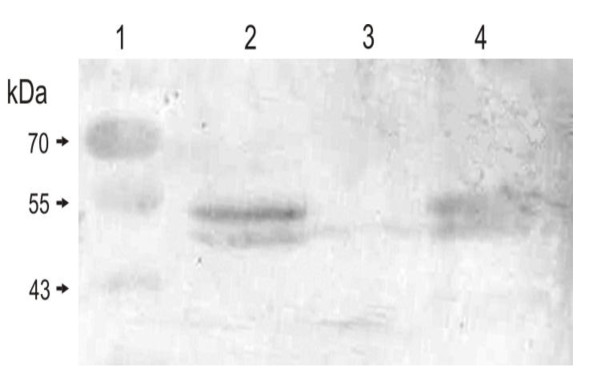
**Expression of *dsbI *from own promoter in *C. jejuni *cells**. Western blot (anti-rDsbI) analysis of *C. jejuni *protein extracts separated by 12% SDS-PAGE. Relative positions of molecular weight markers (lane 1) are listed on the left (in kilodaltons). Lanes 2-4 contain 15 μg of total proteins from: *C. jejuni *81-176 AG6 (Δ*dba-dsbI*)/pUWM1103 (2), AG6 (3) and *C. jejuni *81-176 wt (4)

To further address the role of Dba in *dsbI *expression the recombinant plasmid lacking the *dba *gene but containing the *dsbI *gene transcribed from own promoter was constructed and introduced into the *C. jejuni *81-176 Δ*dba-dsbI::cat *mutant. The *C. jejuni *81-176 Δ*dba-dsbI::cat*, harbouring pUWM769 was employed as a control. Western experiments showed that an individual expression of the *dsbI *gene from own promoter results in DsbI production (Figure 6, lane 2), underlining once more the importance of mRNA secondary structure for the *dsbI *mRNA translation.

## Discussion

The best characterized Dsb oxidative system, that of *E. coli *K-12, consists of two oxidoreductases, periplasmic DsbA and inner membrane DsbB, that are involved in disulfide bond formation *de novo *in the bacterial periplasm. Genes encoding these proteins are located in different chromosomal sites and are transcribed as monocistronic units.

The *Campylobacter jejuni *Dsb oxidative pathway is more complex. In the present study we initiated analysis of *C. jejuni dsb *gene organization and regulation. Our results document organization of these genes in two operons, one comprised of *dba *and *dsbI*, and another of *dsbA2, dsbB *and *astA*. The *dsbA1 *gene constitutes a separate monocistronic transcriptional unit. Predictions based on *in silico *analysis by Petersen *et al*. [44] of the *C. jejuni *NCTC 11168 genome nucleotide sequence stated that the *dba *and *dsbI *genes are cotranscribed. They also indicated that *cj0864 *(a truncated version of *dsbA2*) and *cj0865 *(*dsbB*) potentially form an operon. The first T base of the TATA box was predicted to be located 199 bp upstream from the ATG start codon for the *dba-dsbI *operon and 66 bp from the ATG start codon for the *dsbA2-dsbB-astA *operon [44].

Global comparative *C. jejuni *transcriptome or proteome analysis revealed that transcription levels of *dsbA2, dsbB *and *astA *increase in strains isolated from a chicken cecum compared with strains grown *in vitro *[5] and they are down-regulated under iron-restricted conditions *in vitro *[6]. Stinzi *et al*. found that *dsb *gene transcription was not dependent on the temperature of *in vitro *growth (37 *vs *42°C) [45]. So far only one transcriptomic study has documented that *dba *and *dsbI *transcript abundance is iron-dependent. Interestingly, the authors stated that the transcription of *dba *and *dsbI *was antagonistically regulated by iron accessibility, depending on the experimental conditions, i. e. iron-activated shortly after iron addition into the medium and iron-repressed in the mid-log phase of growth [40]. All cited transcriptomic experiments were conducted on mRNA derived from *C. jejuni *NCTC 11168, a strain which has the shorter, non-functional *dsbA2 *version.

Our experiments, conducted on *C. jejuni *480 wild type expressing β-galactosidase from different *dsb *gene promoters of *C. jejuni *81-176, demonstrated that they are all regulated in response to iron availability. Our data are generally consistent with those derived from transcriptomic analysis. The strongest of the analyzed promoters, P*_dsbA1_*, which was down-regulated in iron starvation conditions, was not identified in comparative transcriptomic experiments conducted by Holmes *et al*., although that work revealed P*_dsbA2dsbBastA _*iron dependence [6]. Such inconsistency of experimental data might be due to limited sensitivity of the transcriptomic strategy previously used. The transcription level of *dsbA1 *is only slightly affected by iron concentration, whereas the transcription level from P*_dsbA2dsbBastA _*decreases about 10-fold in response to iron deficiency. The *dsb *gene promoters are antagonistically regulated by iron availability, at least under conditions used in this study. Thus, abundance of both periplasmic oxidoreductases, DsbA1 and DsbA2, decreases when iron becomes restricted, while DsbB and DsbI membrane oxidoreductases are synthesized constitutively, in different extracellular iron concentrations. This might suggest that iron-storage proteins or non-essential iron-using proteins might be direct or indirect targets of the Dsb oxidative pathway involving activity of DsbA1/DsbB or DsbA2/DsbB redox pairs.

In some microorganisms, positive regulation by Fur and iron is provided by action of sRNAs which are themselves regulated by iron-complexed Fur - these sRNAs pair with their target mRNAs and promote their degradation (reviewed in [46]). However, P*_dsbA2dsbBastA _*and P*_dsbA1 _*promoters are not regulated that way, since the level of β-galactosidase in iron-sufficient medium is comparable in wild-type and *fur *mutated cells. This observation proved that these promoters are not induced by iron-bound Fur, as the level of β-galactosidase expressed from these two fusions is higher in response to iron limitation in the *fur *mutant than in the wild type cells. The most probable explanation of these results is that iron-free Fur is capable of repressing their transcription. Palyada *et al*. [40] performed *in silico *analysis aimed at *Campylobacter *Fur box identification. They inspected 16 DNA fragments located upstream of iron and Fur repressed genes, which allowed them to establish the potential Fur box sequence motif. However, only eleven of the analyzed promoters included this element [40]. So far *C. jejuni's *potential Fur box for apo-Fur repressed genes remains undetermined.

In the present study the EMSA assays confirmed that although all the analyzed promoters were members of the Fur regulon, each of them was regulated by a different mechanism. We showed that both iron-free and iron-complexed Fur can act as a repressor. The observed potential dual regulation of the P*_dsbA2dsbBastA _*promoter, dependent on Fur concentration, still remains unclear. An explanation for this phenomenon requires deeper understanding of the *C. jejuni fur *gene expression. In contrast to *E. coli*, the *C. jejuni fur *gene expression is not autoregulated, and additionally, the iron-responsive Fur regulator of *C. jejuni *is expressed from two separate promoters [47]. Our findings further indicate that transcription under iron-starvation can be controlled by Fur indirectly, as was observed for the *dsbA1 *gene. The sophisticated mechanism regulating *dsb *gene transcription in response to iron availability may be responsible for subtle changes in the abundance and/or activity of various substrates in the Dsb system. We demonstrated that activity of *C. jejuni *81-176 AstA, which is a direct target of Dsb system, is dependent on iron level in the medium. However, as AstA level is dependent on the activities of both DsbA1 and DsbA2 (unpublished results), details of the process remain unclear.

Recently performed comparative *Helicobacter pylori *and *Neisseria gonorrhoeae *transcriptomic analysis also indicated that genes included in the Fur regulon can be positively or negatively regulated in response to iron availability [38,48]. Like *C. jejuni *Fur, *H. pylori *Fur also binds to some promoters in its iron-free form to repress their expression [38,49-51]. *C. jejuni *Fur reveals a relatively high degree of amino acid identity with *H. pylori *Fur. Nonetheless it is not able to complement apo-Fur regulation in an *H. pylori fur *mutant when delivered *in trans *[52]. Such unexpected results might be due to subtle differences in conformation of both proteins. Additional experiments, such as solving the three dimensional structure of *C. jejuni *Fur, are required to clarify the functional differences between Fur proteins of these closely related species. Although both species have AT-rich genomes and some of their promoters have similar structure, it can not be excluded that the *C. jejuni *apo-Fur binding nucleotide sequences are not identical as those determined for *H. pylori *apo-Fur. Also two *H. pylori *promoters, the *pfr *and *sod *gene promoters that are repressed by apo-Fur, exhibited low sequence similarity and revealed different affinities for apo-Fur [38,50].

The second part of our research was aimed at understanding the relationship between *dba *and *dsbI *expression. Experiments employing point mutated *dba *provided evidence for strong translational coupling of the *dba *and *dsbI *genes. Inhibition or premature termination of *dba *mRNA translation resulted in the lack of DsbI. This defect was not complemented by the intact chromosomal *dba *gene in *C. jejuni *81-176 *dsbI::cat*. Translational coupling has already been described and is common among functionally related bacterial genes. It was documented that in many cases it involves operons containing overlapping genes as well as genes constituting an operon and divided by short intergenic region [53,54]. *C. jejuni *81-176 *dba *and *dsbI *do not overlap, but are separated by a relatively short intergenic region (11 bp). Experiments employing a recombinant plasmid that expressed only DsbI verified the importance of the *dba-dsbI *mRNA secondary structure for its translation. Preliminary prediction of the secondary structure for the mRNA region spanning the entire *dba *gene and the 5' end of the *dsbI *gene, indicated that the *dsbI *RBS is located within a stem-loop structure formed by a sequence fragment upstream of the RBS (including the 3' part of the *dba *gene) as well as one downstream of the RBS and spanning the initiator codon of the *dsbI *gene. This suggests that mRNA translation of the *dsbI *gene may be blocked due to the occlusion of the RBS, and that translation of the *dba *mRNA may make the RBS of the *dsbI *gene accessible and hence enable the translation of the *dsbI *gene as well. Verification of this hypothesis requires further analysis.

This coupling mechanism may facilitate interaction between two proteins expressed from the same operon. Data obtained in our study showed that in the absence of Dba, DsbI is intensively degraded in *E. coli *cells. Also in *C. jejuni Δdba-dsbI::cat *cells harboring a recombinant plasmid enabling expression of only DsbI, this protein migrates on SDS-PAGE slightly faster than DsbI produced by wild type cells. It was suggested by *in silico *analysis that the N-terminal domain of DsbI contains five transmembrane helixes and its C-terminal domain achieve a β-propeller structure and localize in the periplasm [18]. DsbI localization in the inner-membrane was documented by a cell fractionation experiment (data not shown). *In silico *prediction also localizes Dba in the IM. Although the specific mechanism of Dba and DsbI interplay is yet unknown, we hypothesize that Dba can act as a periplasmic or transmembrane chaperone, providing the proper folding of the DsbI C-terminal domain, which might be a prerequisite for recruiting other proteins to form an active protein complex.

## Conclusions

The present work documents that iron concentration is a significant factor influencing *dsb *gene transcription. Preliminary results of proteomic experiments aimed at identification of *Campylobacter *Dsb system targets suggest that mutations in *dsb *genes influence the level of a dozen extracytoplasmic proteins (manuscript in preparation). One of them is the periplasmic LivJ protein, which contains four cysteine residues and is involved in the colonization process as shown by Hendrixon and DiRita [55]. Moreover proteomic analysis of iron-regulated *C. jejuni *protein expression done by Holmes *et al*. showed that LivJ abundance is iron-dependent. Because *livJ *gene transcription is not iron nor Fur dependent, most likely the changes in the abundance of this protein are influenced by activity of the Dsb system [6]. Taken together, these results support the notion that iron concentration -through the influence on *dsb *gene expression - might control abundance of the extracytoplasmic proteins during different stages of infection. Our work further shows that the synthesis of the DsbI membrane oxidoreductase is controlled by a translational coupling mechanism. Among bacterial genomes sequenced so far, those of *C. jejuni *strains are extremely compact. About 95% of their content is occupied by protein-coding regions and more than 25% of all genes overlap. Presumably, translational coupling occurs during expression of many other *C. jejuni *operons containing tail-to-head oriented genes with short or no intergenic regions.

## Authors' contributions

ADG conducted out most of the laboratory work. MW and MN, working under supervision of EKJK and ADG, contributed to construction of some transcriptional fusion, mutated *C. jejuni *strains and translational coupling experiments. AML did RT-PCR experiments for the *dba-dsbI *operon as well as expression of *dsbI *from its own promoter, and was involved in drafting the manuscript. RG performed experiments concerning influence of iron concentration on *cjaA *gene expression and AstA activity level. PR performed EMSA assays. AW performed experiments concerning DsbI glycosylation. EKJK conceived the study. EKJK and ADG designed the experiments, and were engaged in data interpretation and drafting the manuscript. All authors read and accepted the final version of the manuscript.

## Supplementary Material

Additional file 1 **Arylsulfatase (AstA) assay in *C. jejuni *81-176 cells**. Arylsulfatase (AstA) activity of *C. jejuni *81-176 cultivated on MH liquid medium under high- and low-iron conditions (chelator) till the culture reached OD_600 _~0,6-0,7. Results are from four assays with each sample performed in triplicate. Values are reported as arylsulphatase units. One unit equals the amount of arylsulfatase required to generate 1 nmol of nitrophenol h^-1 ^per OD_600 _of 1. Click here for file

Additional file 2 **Experiment details concerning DsbI stability and glycosylation**. Click here for file

Additional file 3 **Influence of the *dba*/Dba on DsbI stability in *E. coli *cells**. Western blot (anti-rDsbI) analysis of *C. jejuni/E. coli *protein extracts separated by 12% SDS-PAGE. Relative positions of molecular weight markers (lane 1) are listed on the left (in kilodaltons). Lanes 2-7 contain 20 μg of total proteins from: *C. jejuni *81-176 wt (2), *E. coli*/pBluescript II KS (3), *E. coli*/pUWM453 (*dba-dsbI*) (4), *E. coli*/pUWM454 (*dba*) (5), *E. coli*/pUWM455 (*dsbI*) (6) and *E. coli*/pUWM456 (*dba-dsbI*) (7) Click here for file

Additional file 4 **DsbI glycosylation**. Western blot (anti-rDsbI) analysis of *C. jejuni *protein extracts separated by 12% SDS-PAGE. A **- **proteins isolated from *C. jejuni *81-176 wt and *pglB *isogenic mutant. Relative positions of molecular weight markers (lane 1) are listed on the left (in kilodaltons). Lanes 2 and 3 contain 20 μg of total proteins from: *C. jejuni *81-176 wt (2) and *C. jejuni *81-176 *pglB::cat *(3). B - proteins isolated from *C. jejuni *480 AL4 (*dsbI::cat*) overexpressing DsbI or the mutated version of the protein DsbI. Relative positions of molecular weight markers (lane 1) are listed on the left (in kilodaltons). Lanes 2-4 contain 20 μg of total proteins from: *C. jejuni *480 AL4/pUWM762 (DsbI N292A) (2), AL4/pUWM765 (DsbI N340A) (3) and AL4/pUWM769 (the shuttle plasmid containing a wild type copy of the *C. jejuni dsbI *gene) (4) Click here for file
