# Correction: *Campylobacter jejuni dsb *gene expression is regulated by iron in a Fur-dependent manner and by a translational coupling mechanism

**DOI:** 10.1186/1471-2180-12-58

**Published:** 2012-04-19

**Authors:** Anna D Grabowska, Michał P Wandel, Anna M Łasica, Monika Nesteruk, Paula Roszczenko, Agnieszka Wyszyńska, Renata Godlewska, Elzbieta K Jagusztyn-Krynicka

**Affiliations:** 1 Department of Bacterial Genetics, Institute of Microbiology, University of Warsaw, Miecznikowa 1, 02-096 Warsaw, Poland; 2 Department of Molecular Mechanisms of Mycobacterial Infections, Institute of Pharmacology and Structural Biology, 205, route de Narbonne, 31077 Toulouse cedex, France; 3 Division of Protein and Nucleic Acid Chemistry MRC Laboratory of Molecular Biology, Hills Road, CB2 0QH Cambridge, UK; 4 Department of Gastroenterology, The Medical Centre of Postgraduate Education, Marymoncka 99/103, 01-813 Warsaw, Poland

## Correction

After publication of this work [1], it came to our attention that the grant numbers in the Acknowledgements section were incorrect. This work was supported by two grants from Polish Ministry of Science and Higher Education (No. N303 341835 and N401 183 31/3968) and by intramural grant of University of Warsaw (BW 19126).
