# Supplementary material for: Campylobacter jejuni dsb gene expression is regulated by iron in a Fur-dependent manner and by a translational coupling mechanism
Source: BMC Microbiol. 2011 Jul 25;11:166. doi: 10.1186/1471-2180-11-166 (PMC3167755; doi:10.1186/1471-2180-11-166)
Supplement: Additional file 1 — Arylsulfatase (AstA) assay in C. jejuni 81-176 cells. Arylsulfatase (AstA) activity of C. jejuni 81-176 cultivated on MH liquid medium under high- and low-iron conditions (chelator) till the culture reached OD600 ~0,6-0,7. Results are from four assays with each sample performed in triplicate. Values are reported as arylsulphatase units. One unit equals the amount of arylsulfatase required to generate 1 nmol of nitrophenol h-1 per OD600 of 1. [file 1471-2180-11-166-S1.DOC]

**Additional file 1 - Arylsulfatase (AstA) assay in *C. jejuni*  81-176 cells**

Arylsulfatase (AstA) activity of *C. jejuni* 81-176 cultivated on MH liquid medium under high- and low-iron conditions (chelator) till the culture reached OD_600_ ~0,6-0,7. Results are from four assays with each sample performed in triplicate. Values are reported as arylsulfatase units. One unit equals the amount of arylsulfatase required to generate 1 nmol of nitrophenol h^-1^ per OD_600_ of 1.

|  | **medium** | **OD_410_  (mean value with standard deviation)** | **arylsulfatase units** | **relative activity (%)** |
| --- | --- | --- | --- | --- |
| 1 | + Fe | 0.70 ± 0.006 | 550 | 100 |
|  | + chelator | 0.45 ± 0.008 | 330 | **60.3** |
| 2 | + Fe | 0.74 ± 0.005 | 520 | 100 |
|  | + chelator | 0.49 ± 0.012 | 400 | **76.1** |
| 3 | + Fe | 0.93 ± 0.019 | 720 | 100 |
|  | + chelator | 0.78 ± 0.014 | 540 | **75** |
| 4 | + Fe | 0.58 ± 0.004 | 400 | 100 |
|  | + chelator | 0.45 ± 0.008 | 310 | **78.5** |
