# Supplementary material for: Campylobacter jejuni dsb gene expression is regulated by iron in a Fur-dependent manner and by a translational coupling mechanism
Source: BMC Microbiol. 2011 Jul 25;11:166. doi: 10.1186/1471-2180-11-166 (PMC3167755; doi:10.1186/1471-2180-11-166)
Supplement: Additional file 2 — Experiment details concerning DsbI stability and glycosylation. [file 1471-2180-11-166-S2.DOC]

**Additional file 2 - Experiment details concerning DsbI stability and glycosylation**

**Methods**

**Construct for *C. jejuni* *pglB* mutagenesis**

The 1.2 kb DNA fragment of the *pglB* gene was PCR-amplified from the *C. jejuni* 81-176 chromosome using primer pair: cj1143L1 - cj1431P1 and cloned in the pGEM-T Easy vector. The resulting plasmid was digested with ClaI and HindIII, recognizing sites in the middle of *pglB.* After creating blunt ends using Klenow fragment, plasmid was ligated to the 0.8 kb blunt-ended chloramphenicol resistance cassette excised from pRY109. The resulting suicide plasmid was named pUWM797 (the *cat* cassette inserted in the same transcriptional orientation as *pglB* gene). Inactivated gene version was introduced into the *C. jejuni* 81-176 or 480 chromosome by allele exchange method, as described by Wassenaar *et al.* . Mutants were named respectively WW2 (*C.* *jejuni* 81-176 *pglB::cat*) and WW3 (*C.* *jejuni* 480 *pglB::cat*) (Table 1A).

**Site-directed *C. jejuni dsbI* mutagenesis**

Point mutations were generated using a Quick-Change site-directed mutagenesis kit, following the supplier’s recommendations (Stratagene). To introduce point mutation to plasmid-encoded *dsbI* gene, the pUWM456 was used as a template for PCR-mediated mutagenesis. Point mutations: N292A and N340A were introduced with primers NFS_1 - NFS_2 and NAS_1 - NAS_2, respectively. The mutagenic oligonucleotide primers are summarized in Table 2A. Obtained plasmids, containing the *dsbI* gene encoding protein with single amino acid substitutions, were transformed into *E. coli* DH5α and the presence of the desired mutations verified by DNA sequencing. DNA fragments containing the *C. jejuni* *dsbI* gene with introduced point mutation were cloned into pRY107 shuttle vector using XbaI and SalI restriction enzymes. Resulting plasmids, named pUWM762 (carrying wt *dba*, *dsbI::*N292A) and pUWM765 (carrying wt *dba*, *dsbI::*N340A) were then introduced into AL4 (*dsbI::cat*) by electroporation.

**Results**

***C. jejuni dba-dsbI* expression in *E. coli***

Protein extracts from *E. coli* harbouring recombinant plasmids pUWM453, pUWM454, pUWM455 and pUWM456 (containing *dba-dsbI*, *dba*, *dsbI* and *dba-dsbI,* respectively), were resolved by SDS-PAGE (sodium dodecyl sulfate polyacrylamide gel electrophoresis) and subjected to Western-blotting with rabbit polyclonal specific anti-rDsbI antibodies. Plasmids pUWM456 and pUWM453 differ in inserted *C. jejuni* DNA fragment orientation with respect to the *lacZ* vector gene. In *E. coli*, DsbI undergoes partial degradation, as reflected by many protein forms reacting with specific anti-rDsbI serum (Additional file 3, lane 4 and 7). This effect is more profound when *dba*/Dba is missing (Additional file 3, lane 6).

**Effect of the *pglB* mutation on *C. jejuni* DsbI**

In *C. jejuni* wild type cells the DsbI is produced in two forms, which differ in mobility in SDS-PAG. Mutation of the *pglB* resulted in the loss of the DsbI form of increased mobility, suggesting that DsbI is a target of N-glicosylation process (Additional file 4A).

**Effects of the *dsbI* point mutation on *C. jejuni* DsbI glycosylation**

The amino acid sequence of a periplasm-located DsbI fragment contains four NXS/T N-glycosylation sequences. However, only one of them (290 DNNFS 294) possess a negatively charged amino acid at the position -2 which is a prerequisite for *Campylobacter* N-glycosylation . An asparagine-to-alanine replacement at the position 292 by site-directed mutagenesis resulted in only a nonglycosylated form of DsbI (Additional file 4B, lane 2). In contrast an asparagine-to-alanine mutation at the position 340 (340 NAS 342) did not alter the glycosylation profile of DsbI, and mutated protein was present in SDS-PAG in two forms, as observed for wild type DsbI according to N-glycosylation consensus sequence determined by Kowarik *et al.* (Additional file 4B, lanes 3 and 4) . So far, the role of DsbI glycosylation remains unclear.

**References**

1. Wassenaar TM, Fry BN, van der Zeijst BA: **Genetic manipulation of *Campylobacter*: evaluation of natural transformation and electro-transformation**. *Gene* 1993, **132**(1):131-135.

2. Kowarik M, Young NM, Numao S, Schulz BL, Hug I, Callewaert N, Mills DC, Watson DC, Hernandez M, Kelly JF, *et al*: **Definition of the bacterial N-glycosylation site consensus sequence**. *EMBO J* 2006, **25**(9):1957-1966.

3. Raczko AM, Bujnicki JM, Pawlowski M, Godlewska R, Lewandowska M, Jagusztyn-Krynicka EK: **Characterization of new DsbB-like thiol-oxidoreductases of *Campylobacter jejuni* and *Helicobacter pylori* and classification of the DsbB family based on phylogenomic, structural and functional criteria**. *Microbiology (Reading, England)* 2005, **151**(1):219-23

**Additional Tables**

**Table 1A -** Additional bacterial strains and plasmids used in this study

| **Strain / plasmid** | **Genotype or relevant characteristics** | **Origin** |
| --- | --- | --- |
| **C. jejuni strains** | | |
| WW2 | 81-176 *pglB*::*cat* | This study |
| WW3 | 480 *pglB*::*cat* | This study |
| **Plasmids for mutagenesis** | | |
| pUWM762 | pRY107/ *cjdsbI* (N292A) | This study |
| pUWM765 | pRY107/ *cjdsbI* (N340A) | This study |
| pUWM797 | pGEM-T Easy / *pglB::cat* | This study |
| **Plasmids for translational coupling study** | | |
| pUWM453 | pBluescript II SK / *cjdba-cjdsbI* operon |  |
| pUWM454 | pBluescript II KS / *cjdba* |  |
| pUWM455 | pBluescript II KS / *cjdsbI* |  |
| pUWM456 | pBluescript II KS / *cjdba-cjdsbI* operon |  |

**Table 2A - Oligonucleotides used in this study**

**Bold letters** indicate *C. jejuni* 81-176 sequences. Point mutated nucleotides in primers are marked with **small letters**. Orientation of the primers (**Fwd** states for forward / **Rev** – for reverse) refers to the orientation of particular *C. jejuni* gene studied.

| **Name** | **Sequence** | **Orientation // restriction site** |
| --- | --- | --- |
| cj1143L1 | **ATGCGAAGAATTAGTGGTAGTG** | **Fwd //Ø** |
| cj1431P1 | **GCTCCTGCATGTGATAAAAATCC** | **Rev // Ø** |
| NAS-1 | **GATTTGCAAAAAATCCT**gc**TGCAAGTGAAGAAGATATCGCC** | **Fwd // Ø** |
| NAS-2 | **GGCGATATCTTCTTCACTTGCA**gc**AGGATTTTTTGCAAATC** | **Rev // Ø** |
| NFS-1 | **GGGATGTAGCTTTCTTAGATAAT**gc**TTTTAGCGTTAAAGAAGG** | **Fwd // Ø** |
| NFS-2 | **CCTTCTTTAACGCTAAAA**gc**ATTATCTAAGAAAGCTACATCCC** | **Rev // Ø** |
