# Supplementary material for: Campylobacter jejuni dsb gene expression is regulated by iron in a Fur-dependent manner and by a translational coupling mechanism
Source: BMC Microbiol. 2011 Jul 25;11:166. doi: 10.1186/1471-2180-11-166 (PMC3167755; doi:10.1186/1471-2180-11-166)
Supplement: Additional file 3 — Influence of the dba/Dba on DsbI stability in E. coli cells. Western blot (anti-rDsbI) analysis of C. jejuni/E. coli protein extracts separated by 12% SDS-PAGE. Relative positions of molecular weight markers (lane 1) are listed on the left (in kilodaltons). Lanes 2-7 contain 20 μg of total proteins from: C. jejuni 81-176 wt (2), E. coli/pBluescript II KS (3), E. coli/pUWM453 (dba-dsbI) (4), E. coli/pUWM454 (dba) (5), E. coli/pUWM455 (dsbI) (6) and E. coli/pUWM456 (dba-dsbI) (7) [file 1471-2180-11-166-S3.DOC]

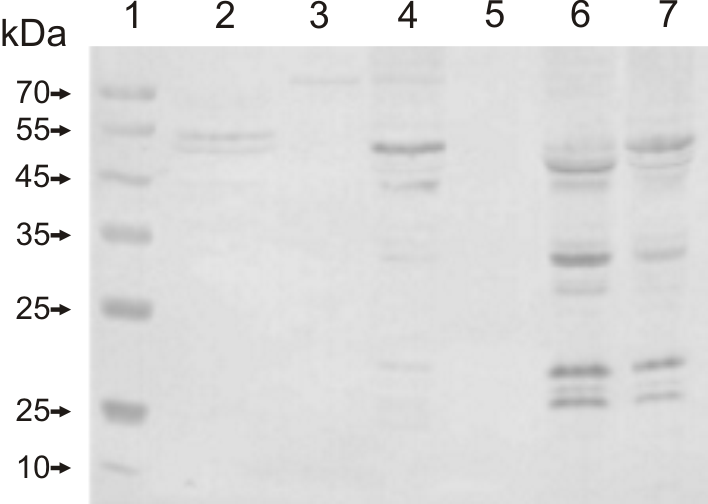


**Additional file 3 - Influence of the *dba*/Dba on DsbI stability in *E. coli* cells**

Western blot (anti-rDsbI) analysis of *C. jejuni/E. coli* protein extracts separated by 12% SDS-PAGE. Relative positions of molecular weight markers (lane 1) are listed on the left (in kilodaltons). Lanes 2-7 contain 20 μg of total proteins from: *C. jejuni* 81-176 wt (2), *E. coli*/pBluescript II KS (3), *E. coli*/pUWM453 (*dba-dsbI*) (4), *E. coli*/pUWM454 (*dba*) (5), *E. coli*/pUWM455 (*dsbI*) (6) and *E. coli*/pUWM456 (*dba-dsbI*) (7)
