# Supplementary material for: Campylobacter jejuni dsb gene expression is regulated by iron in a Fur-dependent manner and by a translational coupling mechanism
Source: BMC Microbiol. 2011 Jul 25;11:166. doi: 10.1186/1471-2180-11-166 (PMC3167755; doi:10.1186/1471-2180-11-166)
Supplement: Additional file 4 — DsbI glycosylation. Western blot (anti-rDsbI) analysis of C. jejuni protein extracts separated by 12% SDS-PAGE. A - proteins isolated from C. jejuni 81-176 wt and pglB isogenic mutant. Relative positions of molecular weight markers (lane 1) are listed on the left (in kilodaltons). Lanes 2 and 3 contain 20 μg of total proteins from: C. jejuni 81-176 wt (2) and C. jejuni 81-176 pglB::cat (3). B - proteins isolated from C. jejuni 480 AL4 (dsbI::cat) overexpressing DsbI or the mutated version of the protein DsbI. Relative positions of molecular weight markers (lane 1) are listed on the left (in kilodaltons). Lanes 2-4 contain 20 μg of total proteins from: C. jejuni 480 AL4/pUWM762 (DsbI N292A) (2), AL4/pUWM765 (DsbI N340A) (3) and AL4/pUWM769 (the shuttle plasmid containing a wild type copy of the C. jejuni dsbI gene) (4) [file 1471-2180-11-166-S4.DOC]

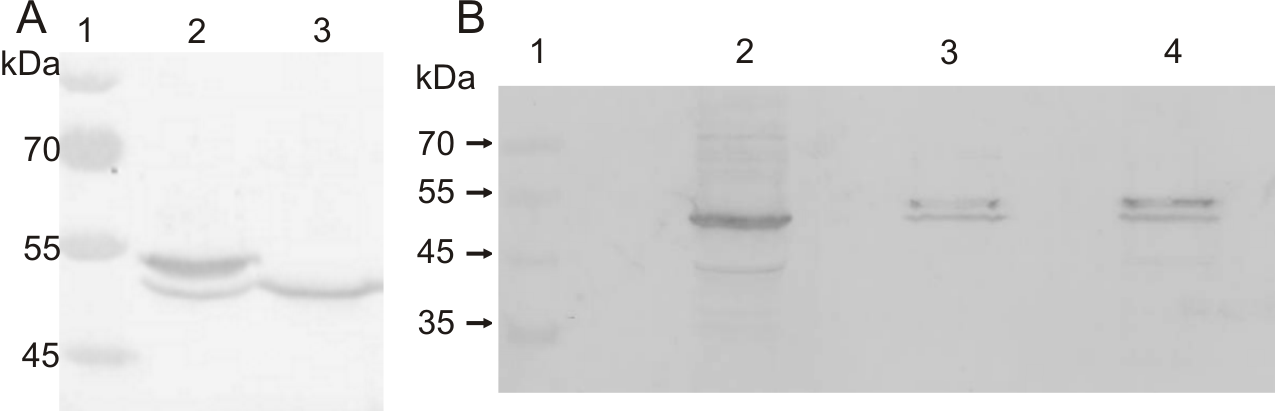


**Additional file 4 - DsbI glycosylation**

Western blot (anti-rDsbI) analysis of *C. jejuni* protein extracts separated by 12% SDS-PAGE.

A **-** proteins isolated from *C. jejuni* 81-176 wt and *pglB* isogenic mutant. Relative positions of molecular weight markers (lane 1) are listed on the left (in kilodaltons). Lanes 2 and 3 contain 20 μg of total proteins from: *C. jejuni* 81-176 wt (2) and *C. jejuni* 81-176 *pglB::cat* (3)

B - proteins isolated from *C. jejuni* 480 AL4 (*dsbI::cat*) overexpressing DsbI or the mutated version of the protein DsbI. Relative positions of molecular weight markers (lane 1) are listed on the left (in kilodaltons). Lanes 2-4 contain 20 μg of total proteins from: *C. jejuni* 480 AL4/pUWM762 (DsbI N292A) (2), AL4/pUWM765 (DsbI N340A) (3) and AL4/pUWM769 (the shuttle plasmid containing a wild type copy of the *C. jejuni* *dsbI* gene) (4)
